# The Control of Environmental Tobacco Smoke: A Policy Review

**DOI:** 10.3390/ijerph6020741

**Published:** 2009-02-20

**Authors:** Aonghus McNabola, Laurence William Gill

**Affiliations:** Department of Civil, Structural and Environmental Engineering, University of Dublin, Trinity College, Ireland; E-Mail: gilll@tcd.ie

**Keywords:** ETS, Smoking Ban, Tobacco Control, Smoking Areas

## Abstract

According to World Health Organisation figures, 30% of all cancer deaths, 20% of all coronary heart diseases and strokes and 80% of all chronic obstructive pulmonary disease are caused by cigarette smoking. Environmental Tobacco Smoke (ETS) exposure has also been shown to be associated with disease and premature death in non-smokers. In response to this environmental health issue, several countries have brought about a smoking ban policy in public places and in the workplace. Countries such as the U.S., France, Italy, Ireland, Malta, the Netherlands, Sweden, Scotland, Spain, and England have all introduced policies aimed at reducing the population exposure to ETS. Several investigations have monitored the effectiveness of these smoking ban policies in terms of ETS concentrations, human health and smoking prevalence, while others have also investigated a number of alternatives to smoking ban policy measures. This paper reviews the state of the art in research, carried out in the field of ETS, smoking bans and Tobacco Control to date and highlights the need for future research in the area.

## 1. Introduction

Globally, tobacco use is associated with five million deaths per annum and is regarded as one of the leading causes of premature death [1]. Compared with non-smokers, smokers are 15 times more likely to develop lung cancer, 11 times more likely to develop chronic lung disease and twice as likely to have acute myocardial infarctions, AMIs, [2]. It is estimated that over 500,000 EU citizens die each year from smoking related ailments [3].

Environmental Tobacco Smoke (ETS, “secondhand smoke”) has been defined as the smoke which non smokers are exposed to when they are in an indoor environment with smokers [4]. ETS has been shown to cause premature death and disease in children and adults who do not smoke, but are passively exposed to ETS [2]. Lung cancer risk has been shown to increase by over 20% with increasing levels of ETS in the workplace [5]. ETS exposure also increases population respiratory symptoms by 30–60% and it is well established that ETS is associated with cardiovascular disease [2]. ETS exposure has also been associated with a 31% increase in the risk of AMIs compared with a doubling of the risk associated with direct smoking [3]. The ingredients in cigarette smoke have been extensively studied and include aluminium phosphate, ammonia, nicotine, colorants, sweeteners and agri-chemical residues. There are also several known carcinogens in the emissions such as lead, benzene, 1,3-butadiene, formaldehyde, mercury and hydrogen cyanide which are frequently used as markers for ETS. More than 4,000 compounds including several toxic volatile organic compounds have been identified in ETS to date [6,7] and it has been declared by the World Health Organisation, as well as by many other independent sources, as carcinogenic [8] with most of these studies also associating ETS with an increased risk of heart disease. ETS has also been shown to have adverse effects on reproduction and cot death in children [8]. Epidemiological evidence has shown that ETS exposure causes an increased risk of cancer of 20–30%, an increased risk of heart disease of 25–30%, an increased risk of strokes of up to 82% and an increased risk of other non-fatal respiratory illnesses [9–11].

Moreover, the risk of the above ailments are high for staff and patrons of bars, restaurants and other hospitality outlets, who are a unique group exposed to extreme levels of ETS, where concentrations of ETS have been shown to be very high [4,12,13], relative to other workers. The combination of cigarette smoke and the drinking of alcohol has also been shown to adversely affect macrophage function [14], thus exacerbating the adverse effects of smoking for the majority of people frequenting public houses. The incidence of such high levels of ETS in public houses and ETS exposure in the workplace has prompted governments around the world to introduce smoking bans and other tobacco control policies in order to reduce its environmental health cost. Banning smoking in indoor public places is regarded as one intervention method to limit ETS exposure among non-smokers, part of a larger effort to reduce tobacco product consumption world-wide, along with nicotine product taxation, adult and children education, and other such approaches [15].

The first smoking ban was attributed to Pope Urban VII in 1590 as he threatened to excommunicate anyone who “took tobacco in the porch-way of, or inside a church, whether it be by chewing, smoking with a pipe or sniffing in powdered form through the nose” [16]. The earliest citywide smoking bans were enacted shortly thereafter in Bavaria, Kursachsen, and certain parts of Austria in the late 1600s. Smoking was banned in Berlin in 1723, in Königsberg in 1742, and in Stettin in 1744 [16]. The first modern, nationwide smoking ban was imposed by the Nazi Party in every university, post office, military hospital and Nazi Party office in Germany, under the Institute for Tobacco Hazards Research, created in 1941 by Adolf Hitler [16]. Major anti-tobacco campaigns were widely broadcast by the Nazis until the demise of the regime in 1945.

In the latter part of the 20th century, research on the risks of ETS began to be made public. This public awareness eventually became public policy in 1975 when the U.S. state of Minnesota enacted the Minnesota Clean Indoor Air Act. This made it the first state to ban smoking in most public spaces (with the exception of bars) and as of October 2007, Minnesota enacted a ban on smoking in all restaurants and bars state-wide, called the Freedom to Breathe Act of 2007. In 1990, the U.S. city of San Luis Obispo, California, became the first city in the world to ban indoor smoking in all public places, including bars and restaurants. The success and resulting popularity of these smoking bans resulted in the implementation of various types of smoking bans in 35 U.S. states. In March 2004, the Irish Government implemented a ban on smoking in the workplace, the first country to do so [4,12]. In Norway similar legislation was put into force in July of the same year. In March 2006, the Scottish Government became the first in the U.K. to implement a smoking ban, which in turn encouraged Wales, Northern Ireland and England to introduce their own legislation [17]. The whole of the U.K. became subject to a ban on smoking in enclosed public places in 2007.

Using the terms ‘smoking ban’ and ‘tobacco control’ as keywords an extensive literature search was carried out in the ScienceDirect, SCOPUS, MedLine and InderScience databases. From the numerous search results a total 63 relevant papers were selected for review. This paper reviews the results of these investigations, into the effects of smoking ban policy on ETS exposure; smoking prevalence; and children. This paper also reviews the evidence of other impacts of smoking bans and of possible alternative policy of tobacco control.

## 2. Smoking Ban Policy and Exposure to ETS

In a study of smoking lounges in California levels of a known marker of ETS, the known carcinogen benzene, were found to range from 3.5 to 14.8 μg/m^3^, depending on the number of cigarettes smoked, volume of the room and ventilation rate [18]. The ETS created by smoking in these environments was found to contribute to up to 60% of the benzene concentration in the room. A further study in Finland into benzene and other VOCs in workplaces where smoking was taking place found levels of benzene in the range 1.0 to 20.2 μg/m^3^, again depending on the number of cigarettes smoked [19]. The recommended annual average limit concentration of benzene, to which an individual is exposed, over a typical lifetime, is 5 μg/m^3^ [20]. These indoor air quality concentrations found as result of the presence of ETS can be clearly seen to greatly exceed the recommended limit value.

In the year 2000, the Tobacco Free Policy Review Group was set up in Ireland to carry out a fundamental review of health and tobacco and make recommendations to the Minister for Health and Children [8]. In line with the recommendations of this report the Office of Tobacco Control (OTC), a statutory body, was set up in May 2002 under the enactment of Section 2 of the Public Health (Tobacco) Act, 2002. This act paved the way for a complete ban on smoking in the workplace, which also included public bars, on March 29^th^ 2004. The banning of smoking in the public bar has received considerable media attention both in other E.U. countries and further a field since it was the first country to affect such a law. The policy was targeted particularly towards reducing the ETS exposure of public bar and other hospitality workers who had, up to then, been exposed to very high concentrations of ETS [4].

A number of investigations were carried out, in which experimental measurements of ETS concentrations were recorded both before and after the implementation of the Irish smoking ban. A study of this nature, which was carried out in nine pubs in Galway (Ireland), found a reduction in PM_2.5_ concentrations of up to 96% and a reduction in PM_10_ concentrations of up to 74% as a result of the smoking ban [12]. Studies of benzene concentrations in two pubs in Dublin (Ireland), following the implementation of a smoking ban have been found to be 0.5 μg/m^3^ on average [12]. This was an average reduction in benzene concentrations of 91% and an average reduction in 1,3-butadiene concentrations of 95% [4]. Both sets of pollutants in these two studies have been shown to have adverse effects on human health [20,21].

Furthermore, modelling investigations into the effect of the Irish smoking ban on the absorption of ETS in the lungs of pub patrons found that the high concentrations of benzene and 1,3-butadiene over a three hour exposure period would potentially be 98% and 100% absorbed into the lungs before the smoking ban, compared to 57% and 65% absorbed into the lungs after the smoking ban was introduced, due to the lower concentrations. Therefore, the large reductions in indoor air quality in pubs in Ireland were also found to be complimented, in terms of health impacts, by a reduction in the percentage of pollutants absorbed into the lungs as a result of lower concentration gradients [4].

Studies have also been carried out in the United States into the effect of smoking bans on ETS exposure. Smoking bans have been introduced in various states and cities in the U.S., without the presence of a nationwide ban as was implemented elsewhere. In Delaware (U.S.), concentrations of particulate matter were reported to have fallen by 90% in public bars and restaurants following the implementation of a smoking ban [13]. The U.S. Center for Disease Control and Prevention carried out investigations of the level of serum cotinine in non smokers during the past decade, finding a reduction of approximately 70% comparing pre and post ban levels [22]. Cotinine is a metabolite of nicotine and is primarily present in non smokers as a result of ETS exposure. In addition 88% of non-smokers in the 3 year period had measurable levels of cotinine in their blood while only 43% had measurable level during 1999 to 2002.

Another investigation of the impact of a smoking ban policy, introduced in Spain on 1^st^ January 2006, found that airborne nicotine levels declined from an average of 0.23 μg/m^3^ before the ban to 0.10 μg/m^3^ after the ban in 44 public hospitals in Spain [23]. In general the study found that ETS in Spanish hospitals decreased although ETS exposure was still prevalent in certain places such as: the main entrance, fire escapes, emergency room waiting areas and cafeterias, explained by the fact that the ban still permits smoking in certain areas (cafeterias & bars). A further investigation of ETS exposure in Spain found that one third of the population were still exposed to ETS in the workplace despite the introduction of the smoking ban [24]. ETS exposure at the entrance to buildings remains problematic in many countries which have ban smoking indoors as smokers tend to go outside the front/back door to smoke, as discussed later in Section 7.

The smoking ban in Scotland, U.K., has been shown to have resulted in a drop in ETS concentrations in bars of 86% [17]. In addition studies have shown 39% reduction in general ETS exposure in Adults and Children in Scotland [25,26]. In Italy, investigations of ETS exposure in two restaurants and two pubs in Milan and six bars in Trieste, following the implementation of a smoking ban, found reductions of 70 to 97% [27,28].

## 3. Smoking Ban Policy and Smoking Prevalence

In addition to reducing the population exposure to ETS and thus improving environmental health smoking ban policy also aims to reduce smoking prevalence, improving the health of smokers. In this regard the policy targets individuals who are ‘social smokers’ and only partake of tobacco use in the social setting of a bar or night club. It also targets those smokers who wish to quit but find it difficult due to the presence of smoking in social outlets such as pubs and night clubs.

**Figure 1.**
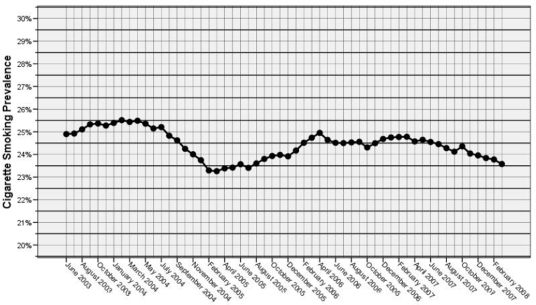
Smoking Prevalence in Ireland June 2003 to February 2008 [31].

Following the implementation of the Irish smoking ban a drop in cigarette sales of 7.5% has been reported in the first six months [4]. In addition, smoking prevalence in the Irish population fell from 27% prior to the ban to 24.1% 8 months following the ban [29]. Irish smoking prevalence has since fallen to 23.6% in March 2008, a 1.2% decrease on smoking prevalence in March 2007 [30]. Figure 1 shows the variation in smoking prevalence over the past 5 years in Ireland, a marked decline in prevalence is notable following the introduction of the smoking ban in March 2004. Smoking prevalence reached its lowest levels in Ireland in February 2005 but subsequently rose in 2006 and is currently declining steadily.

Since the introduction of the Irish smoking ban, smoking prevalence has fallen by approximately 12.5%. However, this smoking ban policy alone cannot claim to be responsible for the entirety of this reduction as cigarette excise duty, anti-tobacco advertising and supports to quit smoking have increased during this period. Equally, in the U.K. as a whole, cigarette sales fell by 11% during July 2007, the first month of the smoking ban in England, compared with July 2006. A survey of 1750 smokers in the U.K., immediately following the implementation of a smoking ban in 2007, found that 1% had quit, and a further 3% intended to quit, in response to the policy measure [32]. In Italy, A systematic review and meta-analysis showed that smoke-free workplaces were associated with reductions in smoking prevalence of 3.8% among employees and with 3.1 fewer cigarettes smoked per day per continuing smoker [33,34]. In Norway, among persons aged 16–74 years in 2003 (prior to their smoking ban) there was a smoking prevalence of 27.3%, which declined to 24.5% in 2006 after the ban [35].

Bans on smoking in restaurants in the United States have also been shown to help stop young people from becoming habitual smokers. A study of youths in Massachusetts found that those in towns with bans were 35 per cent less likely to be habitual smokers [36]. The various smoking ban policies introduced in the U.S. between 1993 and 2003 have been shown to account for about 9% of the decline in adult smoking prevalence during this period [37]. Indeed the same investigation has predicted a further reduction in smoking prevalence in the U.S. of 4.2% should the remaining states without smoking ban policies introduce such laws by 2005. Total prohibition of smoking in the workplace has been found to strongly affect tobacco industry sales volume. Smokers facing these restrictions consume 11%–15% less cigarettes than average and have quit rate that is 84% higher than average [38].

However, in the United States, the Centre for Disease Control has reported a levelling off of smoking rates in recent years despite a large number of ever more severe smoking bans and large tax increases. Anti-smoking groups claim this is due to funding reasons and it has also been suggested that a level of committed smokers has been reached: those unmotivated and increasingly defiant in the face of further anti-tobacco legislation [39].

## 4. Smoking Ban Policy and Public Health

Investigations have also been carried out to quantify the health benefits of smoking ban policy. In the United States a number of investigations have attempted to quantify the effects on cardiovascular health in the population of smoking ban policy, for example in New York, a state-wide ban on smoking was found to reduce ETS exposure, incidence of AMIs and strokes, and a decrease in tobacco consumption [2]. In Helena, Montana, a ban on smoking was introduced in June 2002 and subsequently suspended on legal grounds in December 2002. Helena contained a single hospital which, from 1998 to 2001 and after December 2002, reported 80 AMIs per annum in patients from within the Helena community and 36 AMIs per annum in patients from outside the Helena community. During the 6 month period of the smoking ban, the reported AMIs in patients from within Helena fell to 48 per annum and remained at 36 per annum in patients from outside Helena. This unique set of circumstances showed that for those resident within Helena where the smoking ban was in place AMIs fell by 40%, while for those residents outside Helena AMI rates remained unchanged. In addition the suspension of the smoking ban policy returned AMI rate to pre ban levels.

Similarly, the AMI rates in two hospitals were compared in Colorado, where one hospital served a community which had just introduced a smoking ban and the other had not. The investigation found a decrease in AMIs of 27% in the metropolitan area and 19% in the surrounding county, while no change was found at the other hospital [2].

In other parts of the world, investigations have shown similar trends, following an the introduction of a smoking ban in Scotland, hospital emergency departments have reported a fall in admissions for AMIs of 17% compared with a year on year reduction trend of 3% [40]. In Italy a smoking ban was introduced in January 2005, whereby AMI incidence rates were again reduced as was smoking prevalence and ETS exposure in general [41].

While clear improvements in cardiac health have been shown in numerous studies, the health impacts of smoking ban policy has not generally been found to extend to children. A study of the impact of a smoking ban on asthma related emergency department (ED) visits carried out in the City of Lexington, Kentucky in the U.S. found a 25% decrease in ED visits 32 months after the ban was implement, compared with ED visits 40 months prior to the ban [42]. The study also showed that the pre and post ban asthma related ED visits of children were essentially unaffected. Children with high ETS exposure are predominantly exposed in the home and therefore their exposures are not affected by a ban on smoking bars, public places or the workplace [43]. Studies examining parental smoking bans in the home have found that in households containing non-smokers only that 85% had a total ban on indoor smoking in place. However 50% of households with children and one or more smokers present did not have a full indoor smoking ban in place [44]. Hence, smoking ban policies in the workplace in isolation will not protect the entire population and should be used in combination with other tobacco control initiatives such as taxation, early intervention schemes, education and advertising. Promotion of indoor smoking bans in the home to protect children is a key policy area which needs research and implementation [45].

## 5. Other Impacts of Smoking Ban Policy

In addition to its ineffectiveness in protecting the health of children, smoking ban policies have been shown to have a number of other impacts:

### 5.1. Tourism/Business

Opponents of smoking ban policy such as the alcohol and tobacco industries have carried out numerous investigations which show a negative economic impact on the hospitality industry as a result of the introduction of smoking ban policy [42]. In the United States the spread of smoking ban policy at local, state and national level has slowed as a result of concerns over its economic effects on business, particularly in the hospitality industry. Restaurant and bars owners have raised concerns over possible loss in revenue should smoking be banned on their premises, as they have done in numerous countries around the world in the run up to the implementation of a smoking ban policy [4]. The tobacco industry has fuelled this debate with claims that smoking ban policy will negatively impact on restaurants, bars, nightclubs and other hospitality venues resulting in job and profit losses [42]. Indeed the tobacco industry has a long history of highlighting the dire economic consequences of this and any other tobacco control measures such as increased taxes and bans on tobacco advertising [42,46]. Interestingly, a recent review was carried out evaluating the quality of 97 studies on the economic impacts of smoking ban policies on the hospitality industry. This review found that, of those who reported a negative economic impact on business, 94% of these studies were sponsored by the tobacco industry [47].

In Ireland the alcohol industry, through the Vintners Association of Ireland, mounted the strongest opposition to the introduction of the smoking ban in 2003 and have since claimed it has had negative impacts on businesses in Ireland with the closure of many rural pubs. The Office of Tobacco Control has disputed these claims and has carried out investigations which show no negative effect on business as a result of the ban [29]. Instead findings have shown that the increases in the costs of alcohol and increases in the severity of drink driving legislation, enforcement and penalties, have resulted in rural pub closures and reductions in the pub trade.

Supporters of smoking ban policies have also produced numerous investigations which show a neutral or sometimes positive impact on businesses. A recent review compared the quality and funding source of studies concluding negative economic impacts of smoking ban policy to those concluding no such negative impact [47]. The studies were reviewed independently by two researchers, concluded that all of the best designed studies reported a neutral or positive impact of smoking ban policy on restaurant and bar sales and employment.

The direct medical cost of cardiovascular and other circulatory diseases in the U.S. in 1995 was 151 billion US dollars. Observational studies of the direct medical costs following smoking cessation in observed subjects showed reductions utilisation, which occurred after a lag of three to five years [48]. Therefore showing additional savings to the national economy of participating countries as well as having no negative effects on the tourism/hospitality industries.

### 5.2. Prisons/Psychiatric Hospitals

Sensitive workplaces such as prisons, where every attempt to pacify inmates and protect workers is taken, require special attention when implementing smoking ban policy. Prison officials and guards are often concerned based on previous events in other prisons concerning riots, fostering a cigarette black market within the prison, and other problems resulting from a total prison smoking ban. Prisons have experienced riots when placing smoking bans into effect resulting in prisoners setting fires, destroying prison property, persons being assaulted and injured. A recent example occurred in Quebec (Canada) in February 2008, where a smoking ban was enforced on 18 prisons; the smoking ban was subsequently reversed following rioting by prisoners in these prisons. In Ireland in consultation with the prison service and psychiatric hospitals prior to the implementation of the smoking ban in 2004, both parties felt that the increased health protection afforded to them would be offset against increased risks of inmate attacks, and as a result the smoking ban in Ireland was not extended to these locations.

However, in the U.S. smoking bans have been extended to prisons in many states. Studies have investigated the symptoms of distress and nicotine dependence as predictors of nicotine withdrawal symptoms among incarcerated male smokers during mandated smoking bans [49]. The study concluded that the distressed smokers had the highest level of nicotine withdrawal, indicating that forcing prisoners to quit smoking may not be the best policy for their long term addiction to tobacco and their chances of quitting.

In psychiatric hospitals, the implementation of smoking bans has been treated with similar caution. However, some early investigations have concluded smoking bans can be implemented in psychiatric units without increases in disruption or adverse effects on staff morale [50]. In 1987 a smoking ban was implemented in a psychiatric ward in Washington, U.S., following which an investigation found no change in terms of ward atmosphere, PRN (anti-psychotic) medication usage, and negative incidents [51]. Similarly an investigation in response to staff concerns over a smoking ban at a psychiatric ward in New York, U.S., was carried out, whereby PRN medication, seclusion, restraint, elopement, incident reports and smoking-related discharges were monitored for 232 patients before and after the implementation of the ban. Statistical analysis revealed no significant differences in any of the measured variables following the ban and the study concluded that staff concerns were unfounded [50]. In more recent times an assessment of a partial smoking ban, which was followed by a total smoking ban, in a Swiss hospital was carried out with a view to quantifying the impacts on ETS concentrations and on patient reaction [52]. This investigation found that ETS concentrations decrease after the partial ban on smoking and decreased further after the total ban as would be expected. In addition, among patients, after the total ban, more smokers attempted to quit (18%) compared to before the total ban (2%).

### 5.3. Drink Driving

Increases in fatal accidents as a result of drink driving have been associated with the introduction of smoking bans in the U.S. [53]. Since smoking ban policies in the U.S. have been introduced in various jurisdictions across the country while falling short of an outright national ban, investigations have shown that smokers drive longer distances to bars in other jurisdictions which allow smoking in bars or to other bars within an area under a smoking ban which may facilitate non-compliance or provide outdoor seating. Evidence has shown this behaviour results increased alcohol-related traffic accidents and fatalities. This may be considered an argument against introducing a smoking ban policy or against implementing the policy at a level lower than the nationwide ban which would facilitate this behaviour.

### 5.4. Musical Instruments

Traditional music playing in pubs is commonplace throughout bars in Ireland, Scotland and other parts of the U.K. As a result of the smoking bans introduced in these locations musicians and their instruments are now exposed to considerably lower concentrations of ETS. Musical instruments which are commonly seen in traditional music sessions include the accordion, concertina, melodeon and uilleann pipes, all of which are bellows-driven instruments. As these instruments are played, air from the surroundings is taken in on expansion and expelled on compression of the bellows, as are any pollutants in the air, such as ETS, which is circulated through the instrument in a similar manner.

Anecdotal evidence suggests that the interiors of such instruments, played regularly in smoke-filled environments, become dirty/clogged as a result of the trapping of contaminant particles circulating. Investigators in Ireland conducted a survey of all businesses involved in the repair/cleaning of musical instruments [54]. All participants in the survey encountered a strong smell of cigarette smoke emanating from bellows-driven instruments played in pre-ban smoke-filled environments when they opened; soot-like residue was also deposited throughout the instrument which could be substantial enough to affect the pitch of the instrument. All participants reported a distinct improvement in this situation since the implementation of the smoking ban. In addition, musicians playing wind instruments (for example, flutes and whistles) while exposed to ETS has been shown to be a risk factor in the contraction of lung cancer due to the higher breathing rates required [55]. Indeed a study has shown that individuals exposed to ETS markers such as benzene, breathing at higher rates, absorb more of the pollutant into theirs lungs [56]. Further anecdotal evidence provides examples of several famous traditional Irish musicians who have contracted and died from lung cancer.

## 6. Provision of Smoking Areas

Included in Ireland’s no smoking ban legislation is the provision for employers to create smoking areas for staff and customers provided that they adhere to the design parameters as set out in the legislation. Such smoking areas are then deemed to be exempt from the ban as they are open enough to natural ventilation such that they are effectively considered to be the outdoors. The legislation defines a legal smoking area to be either: *a place or premises that is wholly uncovered by any roof, whether fixed or movable* (Type I); or *an outdoor part of a place or premises covered by a fixed or movable roof, provided that not more than 50% of the perimeter of that part is surrounded by one or more walls or similar structures* (Type II). It is interesting to note that the regulation for the Type II smoking area does not specify any further design requirements (for example, the ratio on the length to breadth of the roof) which has lead to ambiguity, and scenarios where an almost totally enclosed area linked to an open area by an extended roof could still be deemed to comply within the law. A study of smoking areas was carried out in a selection of nine Dublin pubs in 2005 during peak evening hours. Four pubs had Type I smoking areas, four had Type II smoking areas whilst one pub had an illegal smoking area which was effectively a corridor enclosed on all sides. Results for the four pubs with Type I (i.e. uncovered) smoking areas showed mean benzene concentrations of 3.01 and 5.11 μg/m^3^ inside the pub and within the smoking area respectively; similarly, mean butadiene concentrations were 2.44 and 3.56 μg/m^3^ in each respective location. Results for the four pubs with Type II (i.e. covered) smoking areas showed mean benzene concentrations of 1.42 and 5.42 μg/m^3^ inside the pub and within the smoking area respectively; and mean butadiene concentrations of 1.20 and 4.46 μg/m^3^ in each respective location. The pub with the illegal (enclosed) smoking corridor measured mean benzene concentrations of 7.68 and 49.5 μg/m^3^ inside the pub and within the smoking area and mean butadiene concentrations of 3.52 and 60.05 μg/m^3^, respectively. Hence, the more enclosed Type II smoking areas had slightly elevated concentrations at the smoking area (compared to the open Type I area) but seemed to promote lower pollutant concentrations within the pubs. The concentrations in the smoking areas were also strongly related to smoker density, as expected. The indoor pollutant concentrations within the individual pubs were strongly correlated with pollutant concentrations in the smoking areas (most of which were at the pub exit), distance from the smoking area within the pub and the presence of any open windows into the pub in the proximity of the smoking area. These results are also interesting when compared the study carried out in Dublin directly after the smoking ban [4] in pubs without any smoking areas whereby smokers had to stand out on the street which showed benzene and butadiene concentrations of 0.54 and 0.22 μg/m^3^ inside the pub. This was considerably less than the mean value concentrations measured inside the pub from the 8 pubs with formal smoking areas with benzene and butadiene concentrations of 2.22 and 1.65 μg/m^3^. This indicates the negative impact on indoor air quality of locating the smoking areas at the main entrances to the pubs, with open doorways providing a direct link between the generally cooler outside air and warmer air inside the pub, thus creating draughts of the localised polluted air in the smoking area to move indoors.

## 7. Other Tobacco Control Policies and Alternatives to Smoking Bans

Studies have shown that, restrictions on tobacco advertisements, governmental health warnings and taxation on tobacco products, have been successful in decreasing smoking prevalence from 42.4% in 1965 to 24.7% in 1998 in the United States [57]. Tobacco control programs have been introduced in various states in the U.S. since the 1980s, funded largely by tax revenue on cigarette sales. These tobacco control programs have included measures such as: television, radio and print media public education campaigns; school-based tobacco prevention programs; smoking cessation material; telephone ‘quitlines’; policy change and enforcement; *etc*. In the states of Florida, California, Massachusetts and Oregon, where large scale tobacco control programs of this nature have been implemented, these tobacco control programs have been shown to reduce tobacco use [58]. In the period 1990 to 2000 where U.S. tobacco use declined nationally by 20%, in the four states mentioned the rate of decline was an average of 43%.

Excise Duty on cigarette sales is a widely used to reduce tobacco consumption and to generate revenue to fund tobacco control campaigns and to ease the financial burden of smoking-related illnesses and death. Increasing the unit price for tobacco products has been ‘strongly recommended’ as a measure to reduce ETS and tobacco usage, based on strong evidence of its effectiveness, particularly among adolescents and young adults [31]. An empirical investigation on the effectiveness of cigarette taxes on consumption in Taiwan, where the unit cost of cigarettes is very low in comparison to other countries, showed that a 44% increase in unit price would result in a 13% drop in consumption [59]. An investigation comparing the effectiveness of tobacco taxes and anti-smoking advertising campaigns on tobacco consumption, in the United States, found that a 10% increase in tax resulted in a 3% reduction in tobacco consumption [60]. While a 10% increase in anti-tobacco advertising expenditure resulted in a 0.5% decrease in tobacco consumptions. Both measures were shown to be effective but taxation was shown to be the more effective of the two.

The restriction or banning of tobacco advertising has been implemented in many countries as a method of tobacco control as research has shown that tobacco advertising increases tobacco consumption whilst comprehensive tobacco advertising bans reduce consumption and partial advertising bans have little or no effect [61]. It has been shown that a limited set of bans on advertising will not reduce the amount of tobacco advertising expenditure, instead it will be substituted/concentrated into media where the ban is not in operation. When more of the remaining media are eliminated, the options for substitution are also eliminated. An investigation of 22 OECD countries predicted that an introduction of comprehensive bans on tobacco advertising would reduce consumption by over 5% [61].

Opponents to the introduction of a total ban on smoking in the work place, such as the tobacco industry and the hospitality industry, have often cited the installation of efficient ventilation systems as a possible alternative method of reducing ETS exposure concentrations [62]. Ventilation technology falls into two main types: dilution ventilation systems and displacement ventilation systems. Dilution systems work by bringing in fresh air from outside to dilute the concentration of airborne pollutants within the venue – this is by far and away the most common form of ventilation already existing in hospitality venues. Displacement ventilation technology works by supplying fresh air at or near ground level at a low velocity and at a slightly cooler temperature than the ambient indoor air temperature. The cooler air displaces the warmer air (and contaminants) which rise to the ceiling at which point it is exhausted from the room. However, independent investigations have found that while efficient ventilation systems will reduce indoor ETS pollution [63], they have been shown to be an unsatisfactory alternative to a total ban on smoking [8,9]. For example, one investigation into particulate matter concentrations in pubs found that a ventilation rate of up to 400 air changes per hour would be required to reduce concentrations below permissible limits and therefore found ventilations system to be economically unsustainable in achieving the desired reductions in ETS [13]. Indeed the installation of such systems has been shown to be too complex and high in cost to enable widespread implementation [62].

## 8. Discussion and Conclusions

The impacts of smoking ban policies on a number of issues has been identified above and are discussed further below, as are the needs for further research.

### 8.1. Environmental Tobacco Smoke

It is quite clear from the various investigations carried out around the world into the effects of smoking bans on ETS that the policy results in considerable reductions in ambient concentrations, typically in the range of 70–95% depending on the pollutant in question, the volume of the room, and number of cigarettes being smoked. It is also clear that other options such as ventilation systems are unsatisfactory alternatives to the policy in terms of achieving similar ETS reductions and are unsustainable in the present economic/energy conscious climate. However, while ETS concentrations have been shown to be reduced dramatically by smoking ban policies, ETS exposure in pubs, clubs, hospitals and restaurants has been shown to be problematic in certain locations, due to the provision of smoking areas. The location of smoking areas in the doorway of premises or the natural congregation of smokers in these areas, has been shown to lead to elevated ETS exposure concentrations within the premise. Premises which do not provide smoking areas in their main doorway or within their premises have been shown to have considerably lower ambient concentrations of ETS in Ireland. However, only a limited amount of research has been carried out in this area and further research is required to highlight the extent of the problem internationally and to propose measures to address it.

In addition, research has highlighted the failure of smoking ban policy in the protection of children and young adults from ETS exposure as their primary source of ETS is typically in the home. Research is required to identify alternative policies to address this deficiency. Notwithstanding this fact and the problems associated with smoking areas, smoking ban policy can be said to have successfully achieved its primary goal in reducing the exposure of non-smokers to ETS at the various locations around the globe where it has been implemented

### 8.2. Smoking Prevalence

The secondary goal of smoking ban policy, to reduce smoking prevalence and thus improve the health of smokers, has been investigated in different countries, which have generally shown similar downwards trends, with reductions in smoking prevalence of the order of 10% have typically been reported. These initial significant reductions have been attributed to social smokers or those who wish to quit but found the social aspect of smoking difficult to overcome. In the longer term however, some studies have revealed a slight increase in smoking prevalence following the initial drop directly after implementation of the ban, although the net downward trend remains.

In addition to reducing the number of smokers, studies have also identified larger reductions in the sales volumes in the tobacco industry following a smoking ban. Studies have shown greater reductions in cigarette sales volume than smoking prevalence following a ban on smoking. This evidence suggests that smoking ban policy reduces the number of smokers but also reduces the number of cigarettes being smoked among those who still smoke. Therefore, due to the consistent findings of reductions in smoking prevalence and sales volume, the secondary goal of the policy could also be said to be successful where it has been implemented.

### 8.3. Health

The adverse health effects of ETS on humans have been well established particularly in terms of cancer risks and cardiovascular disease. A number of studies have shown decreases in cases of AMI presenting at hospitals serving regions where a smoking ban has been introduced. Reductions in AMIs and emergency room visits of 17 to 25% have been reported in various investigations across the U.S. and Europe. Considering that heart disease is the number one cause of premature death in many countries across the world and is therefore attributed to a very large number of deaths annually, such reductions in AMIs is a considerable endorsement of smoking ban policy. Future research into the economic savings in terms of reduction in health costs versus reduction in cigarette excise duty should also be carried out, which could act as a further stimulus for countries still considering the implementation of such a policy.

### 8.4. The Side Effects

Partial introduction of smoking bans, such as in the United States, compared to nationwide bans have been shown to result in increased incidents of drinking driving fatalities, as has the lack of strict enforcement of the policy. The literature has thus highlighted that best practice, whereby a smoking ban is implemented at the national level and is rigorously enforced, avoids such undesirable side effects.

Impacts of the policy on businesses have been disputed between business interests, promoters of the smoking ban policy and the tobacco industry. However, while there are numerous studies claiming negative impacts and an equal number claiming no negative impact, reviews of this literature have found that to be the most rigorously designed studies, seem to report no negative or positive impacts on business. Those studies which reported the opposite were more likely to have been unscientific in their approach and most were sponsored by the tobacco industry.

Concerns have been raised about the implementation of smoking bans in prison and psychiatric hospitals although their implementation in the latter has generally been shown to be successful in many studies. In prisons however, numerous cases of rioting have been reported as a result of smoking bans and enforced abstinence on smokers has been shown to increase their nicotine withdrawal symptoms and nicotine dependence. Clearly research is still required to highlight alternative policies for prisons to protect the health of prisoners and prison workers alike.

### 8.5. Other Anti-Smoking Policies

Other anti-smoking policy measures such as advertising bans and cigarette taxes have also been shown to be effective in reducing smoking prevalence and ETS. Cigarette taxes were shown to be more effective in achieving reductions than an equivalent increase in anti-tobacco advertising. Furthermore, compared with the reductions achieved by the introduction of smoking bans, both cigarette taxes and advertising normally achieve lower results. However, none of these policies are in competition with one another and can be used together to greatly reduce tobacco usage and ETS exposure.
